# Ethical Principles in Digital Palliative Care for Children: The MyPal Project and Experiences Made in Designing a Trustworthy Approach

**DOI:** 10.3389/fdgth.2022.730430

**Published:** 2022-03-18

**Authors:** Tina Garani-Papadatos, Pantelis Natsiavas, Marcel Meyerheim, Stefan Hoffmann, Christina Karamanidou, Sheila A. Payne

**Affiliations:** ^1^Department of Public Health Policy, School of Public Health, University of West Attica, Athens, Greece; ^2^Centre for Research and Technology Hellas, Thessaloniki, Greece; ^3^Faculty of Medicine, Clinic of Pediatric Oncology and Hematology, Saarland University Hospital and Saarland University, Homburg, Germany; ^4^Serious Games Solutions, a Division of Promotion Software GmbH, Tuebingen, Germany; ^5^International Observatory on End of Life Care, Faculty of Health and Medicine, Lancaster University, Lancaster, United Kingdom

**Keywords:** palliative care, digital health, research ethics, cancer, acceptability, trustworthiness, children

## Abstract

This paper explores the ethical dimension of the opportunity to offer improved electronic patient-reported outcome (ePRO) systems addressing personal needs of pediatric cancer patients, their parents and caregivers, with regard to technological advance of digital health. This opportunity has been explored in the MyPal research project, which aims to assess a patient-centered service for palliative care relying on the adaptation and extension of digital health tools and concepts available from previous projects. Development and implementation of ePROs need to take place in a safe, secure and responsible manner, preventing any possible harm and safeguarding the integrity of humans. To that end, although the final results will be published at the end of the project, this paper aims to increase awareness of the ethical ramifications we had to address in the design and testing of new technologies and to show the essentiality of protection and promotion of privacy, safety and ethical standards. We have thus reached a final design complying with the following principles: (a) respect for the autonomy of participants, especially children, (b) data protection and transparency, (c) fairness and non-discrimination, (d) individual wellbeing of participants in relation to their physical and psychological health status and e) accessibility and acceptability of digital health technologies for better user-engagement. These principles are adapted from the *Ethics Guidelines for a trustworthy Artificial Intelligence (AI)* which provide the framework for similar interventions to be *lawful*, complying with all applicable laws and regulations, *ethical*, ensuring compliance to ethical principles and values and *robust*, both from a technical and social perspective.

## Introduction

Electronic patient-reported outcome (ePRO) systems, namely health outcomes as reported by patients themselves, constitute an important technological advance of digital health which has not yet become an established part of routine healthcare. In view, however, of their potential to significantly improve the patient's Quality of Life (QoL), they are increasingly being established in an innovative way in various health care contexts, such as palliative care (1–3).

This context has been the focus of MyPal, a Horizon 2020 research project (https://mypal-project.eu/ funded by the European Commission) involving a collaboration of 16 clinical, academic and commercial partners. The mutual vision is to explore the role of ePROs in fostering palliative cancer care and in complementing traditional methods of support to cancer patients and to the people who are involved in their care, such as family members, physicians, nurses, psycho-social teams and other health care professionals (HCPs). The consortium's aim has been to design and evaluate a novel ePRO-based digital health platform in a palliative care context, addressing the personal care needs of both adult and pediatric cancer patients, by providing improved ePRO-based tools and functionalities and shifting from passive patient reporting based on conventional non-digital PRO approaches to active digital patient engagement (4). This includes symptom reporting, notifications, educational material and appropriate responses from healthcare providers based on the principles of shared decision making tailored to individual physical and psycho-emotional patient needs (5).

In MyPal, palliative care is understood according to the World Health Organization's (6) definition with the objective to enhance patients' and their families' QoL who are facing serious illness by addressing the problems associated with it *via* a wide spectrum of physical, psychological and spiritual care. This implies to have palliative care being early integrated and not limited only in the terminal stage of serious diseases (7). Early access to palliative care either as a sole treatment or in parallel with routine anticancer treatment, is currently a recommended standard practice for the alleviation of the disease-related aspects (8).

This paper explores the need for increased awareness of the ethical challenges and ramifications of new and advancing technologies which have been addressed in the context of MyPal regarding the intersection of palliative care and digital technologies (1) such as ePROs (9–11). Digital technologies in the research context with the ultimate aim of their integration in routine clinical care (12) and clinical studies raise a number of scientific ethical and data protection challenges (13–17), the latter including the need for development and implementation in a safe, secure and responsible environment, preventing any possible harm and safeguarding the dignity and integrity of participating individuals. At the same time, reliable evidence has to be generated (1, 5).

The focus of the ethical exploration presented in this paper has been the participation of pediatric oncology patients with solid tumors or leukemia, who represent an especially vulnerable group of study participants in an observational prospective clinical feasibility study (MyPal4Kids) which aims to assess the acceptability and appropriateness of the respective developed ePRO-based digital health platform (18). This platform is, after having been validated by end-users, currently in the phase of pilot testing in clinical settings, yielding the first results. The first application designed for child patients comprises a so-called serious game, which combines the motivational character of a non-violent game with the advantages of a modern ePRO-based system (19). The second application implements self and proxy reporting system for parents and health professionals.

## The Ethics of Digital Technologies

The research within the scope of MyPal lies within a rapidly expanding context of mobile Health (mHealth) and electronic Health (eHealth). New definitions of roles and responsibilities have arised, both for patients and physicians (20). According to Topol (21), a prominent cardiologist and proponent of digital health and Artificial Intelligence (AI), the future of health lies in digital technologies. With regard to a recent editorial, *digital medicine is on its way to being just plain medicine* (22). For this reason, development and deployment of similar methodologies, require compliance both with scientific and ethical rules in order to produce benefit for the patients, notwithstanding safety and effectiveness of treatment (20, 23–25).

The need, however, for such a compliance is not self-evident; in fact, it is the historical product of a long journey regarding the relationship of science, technology, ethics and politics (26). This has been comprehensively described by Nydal (27) who has presented three idioms of science, (a) the representational, (b) the performative, and (c) the co-production idiom in the process of the integration of ethics. The *co-production idiom* in particularreflects the view that good science must be intertwined with good politics, demanding appropriate actions.

This need to integrate ethics, is reflected in the conceptual and reference architecture of the MyPal project reflecting the interaction with the world around it. Software architecture is an established term in systems and software engineering, referring to the various architectural styles per category (deployment, communication, structure) and the way they function. But it is also defined as a statement that reflects the concerns of the software stakeholder, whether that be a person, a group or an entity, and where *concerns* refer to a requirement, an objective, an intention, or an aspiration a stakeholder has for that system.

The architecture of the system is analogous to the architecture of a building. The use of this terminology in digital systems, constitutes a metaphor; architecture as an art and process has its own ethical foundations that go beyond the ethical obligations defined in the profession's codes, to the way the design impacts on owners, users and the public and to the way it expresses *how the building speaks* (28). According to Ayiran (29), designing a building which has a certain identity, broadening the feeling, thoughts and imagination of human beings, is a fundamental purpose of architecture for which metaphors are a valuable tool. This metaphor does indeed reflect a landscape beyond mere compliance, a landscape of real reflection and identification of the areas raising potential concern for the rights of the participating children. This concern has been embedded in the MyPal project since its early conception: the consortium involves a number of pediatric oncologists whose primary duty is the welfare of the children under their care. This approach has led to an ethics framework which provides the basis for critical reflection and acts as a tool for addressing the appropriateness of the objectives, the benefit anticipated, the effectiveness of the digital health platform (30), the avoidance of any detriments or burdens, the opposing views and the final implementation in conditions of fairness and transparency (31) as described in the corresponding study protocol (18).

When, however, it comes to practice and implementation, these principles are not as plausible or as easily considered as it seems, raising thus possibilities of undermining the whole process. In this line, Floridi (32) highlights five possible ethical risks in the implementation of digital technologies and describes an environment of proliferation of ethics documents, which may lead to conflicts. However, he keeps a positive stance toward the development of ethics guidelines as they reflect a growing awareness of the ethical ramifications of mobile applications, web-based services and AI.

Studies have shown that serious ethical ramifications in the treatment of pediatric cancer patients mainly concern conflicting perspectives of children, parents and other carers as well as a challenging successful collaboration between all involved parties. The disposition of patients to use digital health apps, their digital literacy level, the burden of the disease and their personal priorities have to be considered. Indeed, technological progress implies besides new ways of communication also new types of data which could potentially provide novel insights to various aspects of a patient's life and facilitate treatment and health care improvement. Simultaneously, the analysis of, e.g., unstructured data such as free text answers, requires providing access to sensitive information to people or software who would not have this access in the realm of non-digitalized healthcare. Such emerging technical paradigms and data sources may disrupt already established healthcare norms, e.g., interaction between patient and HCPs, also in terms of ethics (33). However, the risk of potentially unethical use of these emerging technical paradigms does not eradicate the advantages of potential benefits for the patients, especially when this benefit is clearly outlined (16, 26).

## The Challenge of Normative and Regulatory Compliance

The need for compliance with the regulatory framework, in the context of MyPal4Kids, where innovative digital approaches are implemented by pediatric patients in conditions of vulnerability, suffering and pain, has been an enduring challenge given the potential benefit but also the possible adverse effects on the rights of children. These have been enshrined in various international texts as the result of a normative journey which has provided the theoretical underpinning for health interventions regarding children to be designed and implemented with respect to their dignity and their voice. A number of factors acting both as cause and effect worked in favor of paternalistic and protective attitudes of pediatricians, doubts regarding the comprehensive abilities of the child, limited life experience and traditional assumptions about superiority of adults (34). Eventual changes in the social context, however, affecting family and children were gradually reflected in regulatory attempts to acknowledge the important role of children in society. The Geneva Declaration (35) inspired by the need to support children after the suffering they had been through during the First World War, was adopted by the League of Nations as the first document to recognize that children have specific rights, and in particular *recognizing that mankind owes to the Child the best that it has to give*.

Eventually both scientific knowledge as well as social development led to children being officially acknowledged as a class of their own. Two major legal texts have acted as the basic legally binding pillars toward this protection, the United Nations (UN) Commission on Human Rights' convention on the Rights of the Child (52) and the European Convention on the Exercise of Children's Rights adopted by the Council of Europe (36), the leading Human Rights organization in the European continent. The UN Convention, notwithstanding its special focus on the protection of children in family proceedings before judicial authorities, is based on the notion that children have the right to be informed and the right to express their views, either by themselves or through other persons or bodies (52). Despite the differences of implementation found in national jurisdictions, four provisions of the UN Committee on the Rights of the Child (CRC) were declared as “general principles” essential for the protection of children: non-discrimination, best interests of the child, the right to life survival and development, and the right to be heard. These documents have laid the foreground for the realization of the need to respect children in consideration of their evolving capacities and competencies with regard to the biomedical sciences.

This protection becomes even more necessary within a continuously changing model of heath care and is well reflected in the Guidelines on the Ethics of AI issued by the European Commission (High-Level Expert Group on Artificial Intelligence (AI HLE) (37). The ePRO data collection aims to reveal what is really significant for the patient as very often the significance attributed to a symptom or a side effect by the patient does not always coincide with that of the physician. One of the five key messages of a White Paper (38) on the role of health apps is that a patient-centric health system requires closer partnerships between health professionals, policymakers, health industries, and, obviously, the patient community so that the increasingly complicated interacting system of health care provision also becomes an *eco-system of trust*.

Toward developing trustworthy mobile health apps a mobile health app trustworthiness check list has been created to identify the opinions of the end users on the elements which should characterize a mobile application as trustworthy (39). The list was validated by a sample of persons who in the past 5 years had developed or had been involved in the development of applications aiming to assist end users, including patients with chronic disease. Trustworthiness was investigated in particular with regard to the information provided by the app, organizational attributes, user-friendliness, user control, privacy, access, and the benefit of combining health data from new sources with data collected in the traditional way (e.g., from health and clinical records). The analysis of ePRO-based information provided by the patient can be performed with specific evaluation algorithms and statistics in order to obtain results and draw conclusions that may lead to better treatment. Considering the relation of mobile technology and AI, the European AI Guidelines (37) become very relevant in ensuring a proper level of reflection on various types of challenges. These Guidelines, in exploring compliance with norms and guidelines and contribution to a sustainable environment, delineate three main components of the use of AI: that interventions should be (a) *lawful*, complying with all applicable laws and regulations, (b) *ethical*, ensuring compliance to ethical principles and creating an *ecosystem of trust* by being human-centric, promoting benefit and minimizing harm, and (c) *robust* in a technical and ethical sense.

## Building a Trustworthy Ethical Approach

The use of ePROs will continue to expand in the future including machine learning techniques which will offer higher predictive power with regard to survival and QoL of cancer patients (40). Therefore, compliance with ethical principles in the design and implementation of digital novel technologies is not only a concern of the present but will increasingly be a crosscutting issue in the treatment and empowerment of cancer patients. In this section, we aim to bring together the actual ethical challenges arising in research with a vulnerable population (pediatric patients) and the efforts to operationalize the AI Guidelines for addressing the tension created between the need to balance generation of scientific evidence and respect to human autonomy and privacy (41).

### Respect for Human Autonomy and Human Agency

Respect to individual autonomy is one of the four fundamental ethical principles, developed by Beauchamp and Childress (42), alongside beneficence, non-maleficence and justice. Despite strong critique to the theory of four principles, especially by Clouser and Gert (43), this model remains a fundamental framework extending beyond personal philosophical or political convictions.

Increasing patient self-management and empowerment through the use of applications and/or wearable sensors, has been characterized as one of the *promises* of the mHealth revolution (44). The whole concept of ePROs is based on the importance of the role of the patient's autonomous experience predicated on deliberated self-rule, and the empowerment of patients sought by MyPal reflects how individual autonomy is promoted as patient-generated data, and in particular data on how a child understands and copes with the disease. As the ability of children to report symptoms depends on their verbal skills and developmental level, specific challenges arose with regard to potential use of incorrect terminology, provision of possibly false information and low digital literacy skills; still, on the other hand, clinicians' ability to detect symptoms which have not been reported and psychosocial concerns is also considered as being low (45). To overcome these barriers and to contribute to further improvement of communication with ePROs, our approach was based on a participatory design process where use case scenarios were developed with the participation of all stakeholders in order to properly address and evaluate all relevant concerns of digital literacy and technological barriers. Where necessary, the opinion of focus groups participants was taken into consideration and use cases were revised accordingly. Moreover, previous experience from similar projects like iManageCancer was also adopted (46, 47). As a result, e.g., a questionnaire (48) with age-appropriate versions in terms of wording has been digitally adapted to guarantee a standardized way of reporting symptoms. The presence and severity of symptoms are asked for within small breaks of a violence-free underwater-themed game. The app includes a tutorial and an option to have all texts, questions and answers read aloud, a function incorporated especially to support younger children. Besides, parents as well as clinicians can always answer as proxy for the child.

A number of studies have attempted to evaluate the use of ePROs for cancer patients, either as study end point assessment instruments or as integral intervention components, the former being most often the case than the latter (49). Such studies include the eSMART study, where ePROs aim to enhancing the patient's autonomy via improved self-care efficacy (50) and the LuCApp study, addressed in particular to lung cancer patients and their beneficence, i.e., alerting HCPs if severity of reported symptoms exceeds certain safety limits (51). In MyPal, the following parts (events) of the study in various time points have been designed with special consideration of the autonomy of pediatric patients, revealing an approach in line with the right of the child for autonomy and the right to consent to decisions affecting their life as stipulated in Rights of Children (52).

#### Provision of Information

Provision of information before the enrollment of patients. The child's experience with their care and the stage of their development is being carefully observed in the MyPal4Kids and given the fact that participating children are not legally empowered to decide for themselves, it is important to provide the children with the necessary age-appropriate information to enable them to decide on their participation with tailored messages. Informed consent templates and information sheets were drafted adapted to the level of comprehension of three age groups, a differentiation also based on scientific evidence regarding important variations between different age ranges including a number of aspects which do not appear in a linear way (53).

#### Creation of a Gamified App

Creation of a gamified app for children with cancer, to assess the feasibility of this comprehensive patient-centered approach. Digital games seem to provide a unique tool for enabling patients to exercise some control over their disease, to divert their attention and to help them cope with physical and emotional distress. If properly used by children, these games can have a positive effect on their skills and development as they require quick responses, concentration and imagination (54), toward which the following elements were included in the feasibility assessment:

- Motivating the player to join the underwater “running” game.
- Enhancing the player's ability to customize aspects of the game at any time, as game playing is the prominent functionality of the gamified mobile app.
- Providing the opportunity for the child's own opinion to be heard from inside the game, namely by playing the game and getting occasional questions to which spontaneous symptom reporting must be provided.
- Physically activating the children *via* Augmented Reality and motivating them to collect rewards.

In terms of a beneficial game design, while the technical presentation of the game is out of the current paper's scope, the following key design decisions should be highlighted:

- As the “Game over” concept is typically used in video games and sometimes metaphorically used for death, such a state would not be part of the overall game design.
- As children participating in the study are typically under serious psychological and physical burden, the game should not entail significant effort and as such, it should encourage frequent engagement but only for a restricted amount of time (e.g., 3-5 min sessions).

#### The Right to Withdraw

The right to withdraw from the study has received particular attention in MyPal: the participating child is free to withdraw from the study at any time, a decision which will absolutely not affect any of her rights to receive treatment or other health services. Steps will be taken by the research team to ensure erasure (and not simply deletion) of data from backup and live systems. In some cases, however, according to article 17.3.d of the GDPR exceptions are provided when erasure would be likely to render impossible or seriously impair the process of a study, the person can withdraw but her already collected data will continue to be part of the research albeit in an anonymized format that will fully prevent identification of the person. This exemption is also in compliance with Good Clinical Practice standard (section 4.9.0) as well as with the “Recommendation CM/Rec (2019)2” of the Council of Europe (55), according to which when a subject withdraws from research, their health data processed within the context of this research should be either destroyed or anonymized in a manner which does not compromise the scientific validity of the research. The data subject should be informed accordingly on this option. The Recommendation clearly states that it also *applies in the context of exchange and sharing of health-related data by means of digital tools*. In fact, the very purpose of this Recommendation is to apply the principles enshrined in the Convention for the Protection of Individuals with regard to Automatic Processing of Personal Data (Convention 108), to this new digital environment in order to address the continuous *datafication* of our society. Thus, in this case the parent/legal guardian and the child will be informed and a justified explanation will be provided as well as reassurance about further protection of the child's data. After withdrawal no further data will be collected from this child. In the same line, as it is not always clear whether refraining connotes withdrawal of the participants or whether it means that the child has simply forgotten or neglected to engage with the app, prompters, i.e., reminding messages, should be handled with caution.

#### Protection of Human Agency

Autonomy as a concept is not limited to self-determination: according to Gillon (56), the ability to autonomously formulate views and beliefs and connect them to one's own actions is *tantamount to agency* and this is emphasized in the implementation of AI in health care (57), where respect to human agency constitutes an *enabler* for the person concerned to make autonomous decisions and choices supporting her dignity and freedom. In MyPal this is translated as the need to prevent deception, manipulation and addiction regarding the time consumed in playing, i.e., *game-dosage*. Correlations of more positive outcomes with a specified and predicted duration of play is being investigated (58). In MyPal4Kids this issue has been addressed by enabling the pediatric patient to receive collectables for maximal three gaming sessions per day.

#### Control of One's Self and Limitations

Another aspect with regard to autonomy concerns control of oneself. This should not lead to an overestimation of the application or the interpretation of the system. Thus, what the application aims to do is not to *replace* proper clinical support or intervention but to enhance accurate reporting of facts and symptoms through the patient's own voice (33). A point of clarification also concerns the nature of the MyPal digital health platform which should not be seen as an *emergency* tool since no immediate responses can be provided. On the contrary, in case of an emergency the physician/HCP should be directly contacted outside of the MyPal functionality. Supervision of the interaction of the children (and their parents) with the digital health platform and the apps by HCPs has also been foreseen as limitation.

### Privacy and Protection of Data

This section does not aspire to provide a full description of data protection issues within the project but rather to highlight the principle of privacy and transparency as fundamental components of an ethical framework for the implementation of digital health technologies. According to the WHO (59), “*e-health is the transfer of health resources and health care by electronic means which encompasses the delivery of health information for health professionals and health consumers through the Internet and telecommunications*...”. The expansion of digital health technologies has led to a whole range of new opportunities for the collection of valuable data regarding health care. Compliance with data minimization and transparency requires a balancing between privacy, intrusion and health surveillance and, moreover, a clear distinction between research and clinical data and between information processing and data management conducted in the various clinical sites as part of their typical operations. Especially as far as children are concerned, in compliance with the EU and GDPR rules (Article 8) an advanced layer of protection has been applied with regard to provision of consent, assent and protection of data, deriving from the child's privacy, honor and reputation as fundamental rights of the CRC.

The conceptual framework of the project includes a privacy and security tier, wrapping all other tiers and addressing the privacy of the user and the security of the data through the adoption of the Privacy by Design principles (60). This included a Data Protection Impact Assessment (DPIA) and established measures to enhance privacy (data encryption and anonymization) and integrity of data. The MyPal platform includes local server installations at each clinical site participating in the project and a central installation in the sponsor's site, a structure that clearly defines accessibility issues, as from the local to the central server only synchronized regular anonymized data is transferred.

### Fairness, Diversity, and Non-discrimination

#### Fairness

Fairness has been a necessary feature of a proper design, including equal rights to all participants and avoidance of any type of discrimination. This was especially relevant in the recruitment stage which took place before enrollment for the screening for eligible pediatric cancer patients, according to the inclusion and exclusion criteria defined in the protocol and the recruitment SOP (17). Although these questions seem to be rather methodological in nature, they do implicate an important ethical aspect, as a specific technology should take into consideration potentially unfair and unjustified exclusion of certain categories of individuals.

#### Non-discrimination

Non-discrimination is one of the dimensions of accessibility, besides physical, economic and information accessibility. In the context of digital medicine, non-discrimination also includes avoidance of algorithmic bias in input data and modeling. For this reason, the following were adopted:

- Functionalities which prevent the children from providing random answers
- Choice of game elements that are mainly gender-neutral
- Choice of an art style that is both attractive and motivating for children and adolescents
- Translation of questions in easy wording (e.g. replace the answers with smileys) in order to ensure that the application accommodates the whole range of abilities of the pediatric patients involved, especially of younger ones
- Consideration of the case of children who cannot mobilize e.g. because of neutropenia, post-surgery stages or disease progression
- Establishment of a strategy/set of procedures to avoid creating or reinforcing unfair bias in the symptom prioritization algorithm, both regarding the input data as well as the algorithm design
- Use of available technical tools to improve understanding of data, model and performance
- Consideration of others who may be indirectly affected by the system
- Consideration of language issues which may look diminishing to children, for example the use of the phrase “your feedback as a child” in the information sheets and informed consent forms was considered as inappropriate for the group of adolescents and age-appropriate document versions were created
- A customizable voice-over as read-aloud-function was established in order to support especially younger children with still beginning literacy.

The concepts of *accessibility* and *acceptability* have a long-standing history in healthcare: they are defined in General Comment 14 (61) as two of the four interrelated and essential elements which should be contained in the right to health, understood as the enjoyment of a variety of facilities, goods, services but also as the conditions necessary for the realization of the highest attainable standard of health. Acceptability, or acceptance, in younger populations has been easier to achieve due to a broader exposure of children and adolescents to a number of digital technologies, a fact which has led to their characterization as *digital natives* (62, 63). Still, ensuring the acceptability of digital health technologies for parents and family members providing care is essential in engaging them in the whole research process (3, 61). Therefore, life cycle requirements, confidentiality and improvement of health have been especially relevant for MyPal and the acceptability of and engagement with the introduced digital health platform has been a primary endpoint to be assessed through recording of recruitment, participation and attrition rate as well as collection of qualitative and quantitative data through the System Usability Scale (SUS)2.

In this vein, two steps were taken:

Firstly, information from the participating clinical sites on current care provision and feedback from user groups was collected in order to contribute to the implementation of the platform. The synthesis of the feedback provided (mostly *via* focus groups conducted with patients and their parents) with the initial design, highlighted a number of challenging ethical aspects to be taken into consideration and to be integrated in the process of app gamification.

Secondly, the technology was pilot-tested through end-user workshops to see whether its use was in line with the needs and preferences of the targeted end-users. The reason for this was that one of the ethical challenges of digital technologies is the difference between the intended use of an application and the actual implementation (44) or, in other words, the gap between the intentions of the developer and the actual usage by real people. According to a scoping review (64) some of the aspects which are considered as key factors for the acceptance by users include ease of use, reassurance, high usability and usefulness, correct generation of system alerts and fast response to alerts and convenience of real-time reporting of symptoms. On the other hand, barriers include problems with technology or connectivity, lack of clarity of the language used, generation of false alerts and not least barriers toward the integration of palliative care in children *per se*. In fact, as the latter is being reported as a *structural* barrier in many cases (65), a responsible and ethical approach of digital applications may constitute an important pillar toward the acceptance of palliative care as a core component of comprehensive care and to improved clinical utilization of the information generated.

In relation to acceptability, fairness has been embedded in the participatory process foreseen in which the patient is actively taking part in her own treatment, an element which is found in the WHO definition of eHealth (59). ePROs represent a shift to a patient-centric approach with the aim to increase effectiveness and efficiency by becoming a tool for the expression of the patient's own perspective on the treatment received, the interpretation of the symptoms, the understanding of the situation and the overall QoL the improvement of which is one of the main elements included in definition of eHealth by WHO (59).

### Individual and Social Wellbeing of Participants

The benefit anticipated in this study does not include any interventions to the children, pharmacological or other and does not interfere with the usual care provided, but is expected to occur in addressing both physical and emotional welfare of the participants, as for example in the management of physical and emotional pain: pediatric cancer patients have to deal with frequently painful episodes and reporting of symptoms may prove beneficial as pain is a subjective feeling and provision of relevant *personalized* information may contribute to better management (66). Pain is often surrounded by different scientific approaches regarding specific alleviation treatments (e.g., opioids or benzodiazepines) notwithstanding the major role of the WHO Guidelines on the pharmalogical treatment of persisting pain in children aiming to ensure adequate access to pain management (67). Therefore, individual reactions to suffering are valuable, especially if they are connected to negative expectations produced by pain, which lead to the so-called *pain-related* fear and which create a vicious circle acting as predictor for future pain (68). For this reason, the design has chosen to measure the real distress perceived by children and parents associated with how much each symptom bothered them (rather than burden or satisfaction with care), whether this bean event-like sign (e.g., vomiting) or a more lasting symptom (e.g., nausea). Treatment of pain remains a major issue worldwide, as patients often receive inadequate analgesia. According to the Declaration of Montreal (69) on access to pain management there are *major deficits in knowledge of health professionals regarding the mechanisms and management of pain*, despite the fact that alleviation of pain is acknowledged as a fundamental human right (70, 71).

In the assessment of what is good for children, an initial mapping exercise of the context in which pediatric cancer patients live, took place and the opinion of the medical experts involved was collected though a number of online surveys, to provide the necessary insights of the current palliative care context and practices, as well as the unmet needs of users, especially regarding the participating clinical centers. Input included concept of palliative care, practicalities needed and profile of particular patients in terms of cultural issues.

There is no general agreement on the role of digital tools as the best option to meet a patient's needs. Existing evidence has been characterized as *general* and *inconclusive* (62), by some but others consider digital serious games as an optimal tool for cancer patients (54). Many assumptions are being made on behalf of the patients but further research is required not only regarding technological aspects but also in relation to the ethical and social issues arising (72).

#### Prevention and Mitigation of Harm

The platform developed in MyPal4Kids is a combination of the advantages of a PRO-based system with a gaming aspect *wrapped in an entertaining cover* (17). The role of serious games in improving the QoL has been shown in studies concerning diseases such as chronic diabetes: according to a systematic review, the use of serious games has led to a significant decrease in urgent visits to health units as well as improved self-efficacy (58). Still, according to the WHO (73) youth-centered digital health interventions framework, digital health should not be considered as a silver bullet *per se* but should complement and enhance existing health infrastructure and tools (both digital and non-digital) rather than function as standalone solutions. A potential harm is related to the role of HCP involved as it is often claimed that digital technology will eventually replace professional judgement. Addressing this aspect, the aim has been rather to design studies aiming for the new app interventions to act in a *complementary* way as an add-on to standard care. Prevention of harm was also approached as familiarization with the stage of disease and control of potential disappointment, it was therefore important to take into consideration different levels of physical functioning in relation to the health status of the child and the aims of the game toward better motivation and more activity. Furthermore, it should be emphasized that in order to prevent children from being repetitively asked for irrelevant symptoms, a priority-based algorithm has been developed and validated by the clinical project partners in order to ask symptom-related questions appropriately and according to previously given answers to avoid causing potentially unnecessary psychological burden.

#### Encouragement of the Child's Skill and Avoidance of Deterioration

Encouragement of the child's skill and avoidance of deterioration suffering from cancer may not be only a traumatic experience for children, it may also impact on their developmental and cognitive abilities, comprehension and emotional status. In previous PROs studies, cognitive impairment has been addressed as one of the common symptoms of children with cancer (74), and avoidance of deterioration has already been mentioned with regard to fear-related pain, increased risk of Post Traumatic Syndrome Symptoms (PTSS) and dysfunctions observed in long-term survivors of pediatric cancer (75). Thus, among the various tiers of the system, the tools tier offers a number of functionalities aiming to enable children to fully utilize their potential, knowledge and skills. This includes empowering the child to express her own experience, to participate in handling the disease and to convey subjective information without any type of mediation by a parent or another carer and without any possible tensions or misunderstandings. A number of options for supplying training material were considered such as videos, in-app tutorial, slides handbook, manuals and their advantages and disadvantages were discussed in relation to efficiency but also in relation to the best possible way to ensure the child's right to develop and enhance her cognitive abilities during the course of the illness.

## Conclusions

The thrust of this paper has been the examination of the intersection of novel digital technologies with the care of a vulnerable population in the particular context of palliative care. Therefore, in particular, the established normative framework of ethical principles enshrined in the legal system of Children's Rights have been considered. We have aimed not only to unravel the consecutive challenges faced during the design and the testing of the MyPal system, but also to emphasize that the approach adopted in each of these steps, has been the result of a long-standing ethical culture and of a normative and historical continuum of principles and rights, which were elucidated in this context. Moreover, we showed that they have to be extended in the context of children as participant group in the biomedical sciences and eHealth. In the same token, it is important to remind the right of everyone, including children to enjoy benefits of scientific progress and the corresponding applications. This requires participation of children in research which offers them the opportunity to contribute to results applicable to them, especially as the train of new technical and operational paradigms (Decentralized Clinical Trials, Patient Reported Outcome Measures, Patient Reported Experience Measures, Federated Machine Learning, etc.) is already moving. The application of these approaches in the context of clinical research and clinical practice is not really a question anymore. The question has shifted on “how we are going to integrate ethics” as part of these paradigms' Deployment. However, due to the huge speed of technical developments, their actual use in clinical context defines a constantly “moving target” setting where ethical rules related with the adoption of these technologies need to constantly adapt. The questions arising regarding the sufficiency of international and national law to provide adequate protection for children in relation to biomedicine and the scientific desire to strengthen these rights through technological development and clinical utility provide for us a strong justification for continuous reflection on the actual possibility of implementing ethical principles in practice. Integrating ethical evaluation methodological frameworks as part of the respective technical standards development process could be (at least a part of) the answer to this question as standardization both in the technical and the operational level has been identified as critical and has been widely adopted.

## Author Contributions

TG-P contributed to the ethical analysis and wrote the first draft of the manuscript. PN contributed to the technological part of the paper. MM provided input on the feasibility study aspects. SH provided input on the gamified application aspects. CK contributed to the ethical and social analysis. SP reviewed the whole manuscript and provided input with regard to palliative care aspects. All authors contributed to manuscript revision, read, and approved the submitted version.

## Funding

MyPal: Fostering Palliative Care of Adults and Children with Cancer through Advanced Patient Reported Outcome Systems, was funded by the Horizon 2020 Framework Programme of the European Union under Grant Agreement Nr. 825872.

## Conflict of Interest

SH is employed by Serious Games Solutions, a division of promotion Software GmbH, Tuebingen, Germany. The remaining authors declare that the research was conducted in the absence of any commercial or financial relationships that could be construed as a potential conflict of interest.

## Publisher's Note

All claims expressed in this article are solely those of the authors and do not necessarily represent those of their affiliated organizations, or those of the publisher, the editors and the reviewers. Any product that may be evaluated in this article, or claim that may be made by its manufacturer, is not guaranteed or endorsed by the publisher.

## References

[1] Archer S Cheung N Williams I Darzi A. The impact of digital health interventions on the psychological outcomes of patients and families receiving paediatric palliative care: a systematic review and narrative synthesis. Palliat Med. (2021) 35:2017–23. 10.1177/02692163211026523 34159851 PMC8641035

[2] Karamanidou C Natsiavas P Koumakis L Marias K Schera F Schäfer M. Electronic patient-reported outcome-based interventions for palliative cancer care: a systematic and mapping review. JCO Clin Cancer Inform. (2020) 4:647–56. 10.1200/CCI.20.00015 32697604 PMC7397776

[3] Widberg C Wiklund B Klarare A. Patients' experiences of eHealth in palliative care: an integrative review. BMC Palliat Care. (2020) 19:158. 10.1186/s12904-020-00667-1 33054746 PMC7560207

[4] Moerenhout T Devisch I Cornelis GC. E-health beyond technology: analyzing the paradigm shift that lies beneath. Med Health Care Philos. (2018) 21:31–41. 10.1007/s11019-017-9780-3 28551772

[5] Schneeweiss S Eichler HG Garcia-Altes A Chinn C Eggimann AV Garner S. Real world data in adaptive biomedical innovation: a framework for generating evidence fit for decision-making. Clin Pharmacol Ther. (2016) 100:633–46. 10.1002/cpt.512 27627027

[6] World Health Organization (WHO). Palliative Care - Key Facts. (2020). Available online at: https://www.who.int/news-room/fact-sheets/detail/palliative-care (accessed June 22, 2021).

[7] World Health Assembly (WHA). WHA67.19 Strengthening of palliative care as a component of comprehensive care throughout the life course. In Sixty-seventh World Health Assembly. (2014). Available online at: https://apps.who.int/iris/bitstream/handle/10665/162863/A67_R19-en.pdf?sequence=1andisAllowed=y (accessed June 22, 2021).

[8] Coelho A Parola V Cardoso D Bravo ME Apóstolo J. Use of non-pharmacological interventions for comforting patients in palliative care: a scoping review. JBI Database Syst Rev Implement Rep. (2017) 15:1867–904. 10.11124/JBISRIR-2016-003204 28708751

[9] Bennett AV Jensen RE Basch E. Electronic patient-reported outcome systems in oncology clinical practice. CA Cancer J Clin. (2012) 62:337–47. 10.3322/caac.21150 22811342

[10] Preston N van Delden JJ Ingravallo F Hughes S Hasselaar J van der Heide A. Ethical and research governance approval across Europe: experiences from three European palliative care studies. Palliat Med. (2020) 34:817–21. 10.1177/0269216320908774 32186242 PMC7521003

[11] Payne SA Moore DC Stamatopoulos K. MyPal: designing and evaluating digital patient-reported outcome systems for cancer palliative care in Europe. J Palliat Med. (2021) 24:962–4. 10.1089/jpm.2021.0120 34128712

[12] Biesdorf S Biedermann F. Healthcare's Digital Future. (2014). Available online at: https://www.mckinsey.com/industries/healthcare-systems-and-services/our-insights/healthcares-digital-future (accessed June 22, 2021).

[13] Bartholdson C Lützén K Blomgren K Pergert P. Experiences of ethical issues when caring for children with cancer. Cancer Nurs. (2015) 38:125–32. 10.1097/NCC.0000000000000130 24945260

[14] Doyle-Lindrud S. State of eHealth in cancer care: review of the benefits and limitations of ehealth tools. Clin J Oncol Nurs. (2020) 24:10–5. 10.1188/20.CJON.S1.10-15 32441698

[15] Guo C Ashrafian H Ghafur S Fontana G Gardner C Prime M. Challenges for the evaluation of digital health solutions-A call for innovative evidence generation approaches. NPJ Digital Med. (2020) 3:110. 10.1038/s41746-020-00314-2 32904379 PMC7453198

[16] Meirte J Hellemans N Anthonissen M Denteneer L Maertens K Moortgat P. Benefits and disadvantages of electronic patient-reported outcome measures: systematic review. JMIR Perioper Med. (2020) 3:e15588. 10.2196/15588 33393920 PMC7709853

[17] Meyerheim M Burns-Gebhart A Mirzaie K Garani-Papadatos T Braun Y Graf N. Challenges and pitfalls for implementing digital health solutions in clinical studies in Europe. Front Digital Health. (2021) 3:730680. 10.3389/fdgth.2021.730680 34713200 PMC8521796

[18] Meyerheim M Karamanidou C Payne S Garani-Papadatos T Sander A Downing J. MyPal-child study protocol: an observational prospective clinical feasibility study of the MyPal ePRO-based early palliative care digital system in paediatric oncology patients. BMJ Open. (2021) 11:e045226. 10.1136/bmjopen-2020-045226 33849855 PMC8051393

[19] Hoffmann S Schraut R Kröll T Scholz W Belova T Erhardt J. AquaScouts: ePROs implemented as a serious game for children with cancer to support palliative care. Front Digital Health. (2021) 3:730948. 10.3389/fdgth.2021.730948 34957461 PMC8692290

[20] Lau N Waldbaum S Parigoris R O'Daffer A Walsh C Colt SF. eHealth and mHealth psychosocial interventions for youths with chronic illnesses: systematic review. JMIR Pediatr Parent. (2020) 3:e22329. 10.2196/22329 33075743 PMC7685926

[21] Topol E. A discussion with digital health pioneer Dr. Eric Topol. Interview by Paul Webster. CMAJ. (2013) 185:E597. 10.1503/cmaj.109-4546 23922339 PMC3778488

[22] Steinhubl SR Topol EJ. Digital medicine, on its way to being just plain medicine. NPJ Digital Med. (2018) 1:20175. 10.1038/s41746-017-0005-1 31304349 PMC6550249

[23] Akard TF Dietrich MS Friedman DL Hinds PS Given B Wray S. Digital storytelling: an innovative legacy-making intervention for children with cancer. Pediatr Blood Cancer. (2015) 62:658–65. 10.1002/pbc.25337 25586983 PMC4339662

[24] Calvert M Thwaites R Kyte D Devlin N. Putting patient-reported outcomes on the Big Data Road Map. J Royal Soc Med. (2015) 108:299–303. 10.1177/0141076815579896 25827908 PMC4535436

[25] Bird M Li L Ouellette C Hopkins K McGillion MH Carter N. Use of synchronous digital health technologies for the care of children with special health care needs and their families: scoping review. JMIR Pediatr Parenting. (2019) 2:e15106. 10.2196/15106 31750840 PMC6895870

[26] Vayena E Haeusermann T Adjekum A Blasimme A. Digital health: meeting the ethical and policy challenges. Swiss Med Weekly. (2018) 148:w14571. 10.4414/smw.2018.14571 29376547

[27] Nydal R. Why is integration so difficult? Shifting roles of ethics and three idioms of thinking about science, technology and society. Etikk i praksis-Nordic J Appl Ethics. (2015) 9:21–36. 10.5324/eip.v9i1.1835

[28] DuBois C. The Ethics of Architectural Practice (2021). Available online at: https://www.aia.org/articles/1166-the-ethics-of-architectural-practice (accessed June 22, 2021).

[29] Ayiran N. The role of metaphors in the formation of architectural identity. ITU A|Z J Faculty Architect. (2012) 9:1–21. Retrieved from https://www.journalagent.com/itujfa/pdfs/ITUJFA_9_2_1_21.pdf (accessed February 14, 2022).

[30] Granja C Janssen W Johansen MA. Factors determining the success and failure of ehealth interventions: systematic review of the literature. J Med Internet Res. (2018) 20:e10235. 10.2196/10235 29716883 PMC5954232

[31] Kass NE. An ethics framework for public health and avian influenza pandemic preparedness. Yale J Biol Med. (2005) 78:239–254. 17132331 PMC2259154

[32] Floridi L. Translating principles into practices of digital ethics: five risks of being unethical. Philos Technol. (2019) 32:185–93. 10.1007/s13347-019-00354-x

[33] Payne S Tanner M Hughes S. Digitisation and the patient–professional relationship in palliative care. Palliat Med. (2020) 34:441–3. 10.1177/0269216320911501 32253996

[34] Rylance G. Making decisions with children. BMJ. (1996) 312:794. 10.1136/bmj.312.7034.794 8608276 PMC2350696

[35] *The Geneva Declaration of the Rights of the Child of 1924 - UN Documents: Gathering a Body of Global Agreements*. (2021). Available online at: http://www.un-documents.net/gdrc1924.htm (accessed June 22, 2021).

[36] Council of Europe. European convention on the exercise of children's rights. Int Legal Materials. (1996) 35:651–9. 10.1017/S0020782900024244

[37] High-Level Expert Group on Artificial Intelligence (AI HLE). Ethics Guidelines for trustworthy AI. Report Commissioned by the European Commission. (2019). Available online at: https://digital-strategy.ec.europa.eu/en/library/ethics-guidelines-trustworthy-ai (accessed January 24, 2022).

[38] White Paper. Health apps - patients, standards policies. Based on a seminar held at the King's Fund, London, 28 October 2013. (2014). Available online at: http://alexwyke.wordpress.com/2014/03/17/white-paper-health-apps-where-do-they-make-sensewhite-paper-health-apps-from-the-perspectives-of-patients-standards-and-policies-based-on-a-seminar-held-at-the-kings-fund-london-28-oc/ (accessed June 23, 2021).

[39] van Haasteren A Vayena E Powell J. The mobile health app trustworthiness checklist: usability assessment. JMIR mHealth uHealth. (2020) 8:e16844. 10.2196/16844 32706733 PMC7404005

[40] Lehmann J Cofala T Tschuggnall M Giesinger JM Rumpold G Holzner B. Machine learning in oncology—perspectives in patient-reported outcome research. Onkologe. (2021) 27(Suppl 2):S150–5. 10.1007/s00761-021-00916-9

[41] Townend D. Conclusion: harmonisation in genomic and health data sharing for research: an impossible dream?. Hum Genet. (2018) 137:657–64. 10.1007/s00439-018-1924-x 30120573 PMC6132652

[42] Beauchamp TL Childress J. Principles of Biomedical Ethics. 1st ed. New York, NY:Oxford University Press (2001) 1979:5th ed, 2001.

[43] Clouser KD Gert B. A critique of principlism. J Med Philos. (1990) 15:219–36. 10.1093/jmp/15.2.219 2351895

[44] Lucivero F Jongsma KR. A mobile revolution for healthcare? Setting the agenda for bioethics. J Med Ethics. (2018) 44:685–9. 10.1136/medethics-2017-104741 29907579 PMC6173811

[45] Baggott C Baird J Hinds P Ruland CM Miaskowski C. Evaluation of sisom: A computer-based animated tool to elicit symptoms and psychosocial concerns from children with cancer. Eur J Oncol Nurs. (2015) 19:359–69. 10.1016/j.ejon.2015.01.006 25691300

[46] Busse TS Jux C Kernebeck S Dreier LA Meyer D Zenz D. Needs assessment for the development of an electronic cross-facility health record (ECHR) for pediatric palliative care: a design thinking approach. Children. (2021) 8:602. 10.3390/children8070602 34356581 PMC8304612

[47] Kondylakis H Bucur A Crico C Dong F Graf N Hoffman S. Patient empowerment for cancer patients through a novel ICT infrastructure. J Biomed Inform. (2020) 101:103342. 10.1016/j.jbi.2019.103342 31816400

[48] Dupuis LL Johnston DL Baggott C Hyslop S Tomlinson D Gibson P. Validation of the symptom screening in pediatrics tool in children receiving cancer treatments. J Natl Cancer Instit. (2018) 110:661–8. 10.1093/jnci/djx250 29272441 PMC6005103

[49] Leahy AB Schwartz LA Li Y Reeve BB Bekelman JE Aplenc R. Electronic symptom monitoring in pediatric patients hospitalized for chemotherapy. Cancer. (2021) 127:2980–9. 10.1002/cncr.33617 33945640 PMC9020204

[50] Maguire R Fox PA McCann L Miaskowski C Kotronoulas G Miller M. The eSMART study protocol: a randomised controlled trial to evaluate electronic symptom management using the advanced symptom management system (ASyMS) remote technology for patients with cancer. BMJ Open. (2017) 7:e015016. 10.1136/bmjopen-2016-015016 28592577 PMC5734219

[51] Ciani O Cucciniello M Petracca F Apolone G Merlini G Novello S. Lung Cancer App (LuCApp) study protocol: a randomised controlled trial to evaluate a mobile supportive care app for patients with metastatic lung cancer. BMJ Open. (2019) 9:e025483. 10.1136/bmjopen-2018-025483 30772862 PMC6398637

[52] UN Commission on Human Rights (CHR). Convention on the Rights of the Child. E/CN.4/RES/1990/74. UN Commission on Human Rights (1990).

[53] Zillén K Garland J Slokenberga S. The Rights of Children in Biomedicine: Challenges Posed by Scientific Advances Uncertainties. Uppsala University. Report Commissioned by the Committee on Bioethics (DH-BIO) for the Council of Europe. (2017). Available online at: https://rm.coe.int/16806d8e2f (accessed June 22, 2021).

[54] Ghazisaeidi M Safdari R Goodini A Mirzaiee M Farzi J. Digital games as an effective approach for cancer management: opportunities and challenges. J Educ Health Promot. (2017) 6:30. 10.4103/jehp.jehp_146_14 28584830 PMC5441205

[55] Council of Europe. Protection of Health-Related Data - Recommendation CM/Rec(2019)2. Adopted by the Committee of Ministers of the Council of Europe. (2019). Available online at: https://edoc.coe.int/en/international-law/7969-protection-of-health-related-date-recommendation-cmrec20192.html (accessed January 24, 2022).

[56] Gillon R. Introduction. In: Gillon R editor. Principles of Health Care Ethics. Chichester: John Wiley and Sons (1994).

[57] Cohen IG Evgeniou T Gerke S Minssen T. The European artificial intelligence strategy: implications and challenges for digital health. Lancet Digital Health. (2020) 2:e376–9. 10.1016/S2589-7500(20)30112-6 33328096

[58] Holtz BE Murray K Park T. Serious games for children with chronic diseases: a systematic review. Games Health J. (2018) 7:291–301. 10.1089/g4h.2018.0024 29957082

[59] World Health Organization (WHO). From Innovation to Implementation – eHealth in the WHO European Region. (2016). Available onlien at: https://www.euro.who.int/_data/assets/pdf_file/0012/302331/From-Innovation-to-Implementation-eHealth-Report-EU.pdf (accessed June 22, 2021).

[60] Cavoukian A. Privacy by Design: The 7 Foundational Principles. vol. 5. Information and Privacy Commissioner of Ontario (2009).

[61] UN Committee on Economic Social and Cultural Rights (CESCR). General Comment No. 14: The Right to the Highest Attainable Standard of Health (Art. 12 of the Covenant). E/C.12/2000/4. UN Committee on Economic Social and Cultural Rights (CESCR) (2000).

[62] Cheng L Duan M Mao X Ge Y Wang Y Huang H. The effect of digital health technologies on managing symptoms across pediatric cancer continuum: a systematic review. Int J Nurs Sci. (2020) 8:22–9. 10.1016/j.ijnss.2020.10.002 33575441 PMC7859551

[63] Pathipati AS Azad TD Jethwani K. Telemedical education: training digital natives in telemedicine. J Med Internet Res. (2016) 18:e193. 10.2196/jmir.5534 27405323 PMC4961876

[64] Aapro M Bossi P Dasari A Fallowfield L Gascón P Geller M. Digital health for optimal supportive care in oncology: benefits, limits, future perspectives. Support Care Cancer. (2020) 28:4589–612. 10.1007/s00520-020-05539-1 32533435 PMC7447627

[65] Ehrlich BS Movsisyan N Batmunkh T Kumirova E Borisevich MV Kirgizov K. Barriers to the early integration of palliative care in pediatric oncology in 11 Eurasian countries. Cancer. (2020) 126:4984–93. 10.1002/cncr.33151 32813913 PMC7981844

[66] Tutelman PR Chambers CT Stinson JN Parker JA Fernandez CV Witteman HO. Pain in children with cancer: prevalence, characteristics, parent management. Clin J Pain. (2018) 34:198–206. 10.1097/AJP.0000000000000531 28678061

[67] World Health Organization (WHO). Persisting Pain in Children Package: WHO Guidelines on the Pharmacological Treatment of Persisting Pain in Children With Medical Illnesses. World Health Organization (2012). Available online at: https://apps.who.int/iris/handle/10665/44540 (accessed June 22, 2021). 23720867

[68] Boersma K Linton SJ. Expectancy, fear and pain in the prediction of chronic pain and disability: a prospective analysis. Eur J Pain. (2006) 10:551–7. 10.1016/j.ejpain.2005.08.004 16199189

[69] International Pain Summit of the International Association for the Study of Pain (IASP). Declaration of Montréal: declaration that access to pain management is a fundamental human right. J Pain Palliat Care Pharmacother. (2011) 25:29–31. 10.3109/15360288.2010.547560 21426215

[70] Brennan F Carr DB Cousins M. Pain management: a fundamental human right. Anesth Analgesia. (2007) 105:205–21. 10.1213/01.ane.0000268145.52345.55 17578977

[71] Knaul FM Farmer PE Krakauer EL De Lima L Bhadelia A Jiang Kwete X. Alleviating the access abyss in palliative care and pain relief-an imperative of universal health coverage: the Lancet Commission report. Lancet. (2018) 391:1391–454. 10.1016/S0140-6736(17)32513-8 29032993

[72] Lehoux P Gauthier P Williams-Jones B Miller FA Fishman JR Hivon M. Examining the ethical and social issues of health technology design through the public appraisal of prospective scenarios: a study protocol describing a multimedia-based deliberative method. Implement Sci. (2014) 9:81. 10.1186/1748-5908-9-81 24952582 PMC4229879

[73] World Health Organization (WHO). Youth-Centred Digital Health Interventions: A Framework for Planning, Developing and Implementing Solutions With and for Young People. World Health Organization (?2020)?. Available online at: https://apps.who.int/iris/handle/10665/336223 (accessed June 22, 2021).

[74] Meryk A Kropshofer G Hetzer B Riedl D Lehmann J Rumpold G. Implementation of daily patient-reported outcome measurements to support children with cancer. Pediatr Blood Cancer. (2021) 68:e29279. 10.1002/pbc.29279 34383360

[75] Seitz DC Knaevelsrud C Duran G Waadt S Loos S Goldbeck L. Efficacy of an internet-based cognitive-behavioral intervention for long-term survivors of pediatric cancer: a pilot study. Support Care Cancer. (2014) 22:2075–83. 10.1007/s00520-014-2193-4 24639036

